# Bacteria as Bio-Template for 3D Carbon Nanotube Architectures

**DOI:** 10.1038/s41598-017-09692-2

**Published:** 2017-08-29

**Authors:** Sehmus Ozden, Isaac G. Macwan, Peter S. Owuor, Suppanat Kosolwattana, Pedro A. S. Autreto, Sushila Silwal, Robert Vajtai, Chandra S. Tiwary, Aditya D. Mohite, Prabir K. Patra, Pulickel M. Ajayan

**Affiliations:** 1 Materials Physics and Applications Division, Los Alamos National Laboratory, Los Alamos, NM 87545 USA; 2 Department of Biomedical Engineering, University of Bridgeport, 126 Park Avenue, Bridgeport, CT 06604 USA; 3 Department of Material Science and NanoEngineering, Rice University, Houston, Texas 77005 USA; 4 Universidade Federal do ABC, Santo André-SP, 09210-580 Brazil

## Abstract

It is one of the most important needs to develop renewable, scalable and multifunctional methods for the fabrication of 3D carbon architectures. Even though a lot of methods have been developed to create porous and mechanically stable 3D scaffolds, the fabrication and control over the synthesis of such architectures still remain a challenge. Here, we used Magnetospirillum magneticum (AMB-1) bacteria as a bio-template to fabricate light-weight 3D solid structure of carbon nanotubes (CNTs) with interconnected porosity. The resulting porous scaffold showed good mechanical stability and large surface area because of the excellent pore interconnection and high porosity. Steered molecular dynamics simulations were used to quantify the interactions between nanotubes and AMB-1 via the cell surface protein MSP-1 and flagellin. The 3D CNTs-AMB1 nanocomposite scaffold is further demonstrated as a potential substrate for electrodes in supercapacitor applications.

## Introduction

Even though there are a lot of reports on fabrication of three-dimensional (3D) carbon-based architectures and their applications, it is needed that such 3D CNT structures are developed through green and facile methods for the production of porous, light-weight and robust 3D architectures. Fabrication of 3D carbon-based materials, such as carbon nanotube sponge^1–3^, graphene foam^4, 5^, and carbon aerogels^6, 7^, using a variety of approaches is attracting high interest because of the ability to tune their physical properties such as density, mechanical properties, porosity and so on. Novel 3D scaffolds have been developed using solution chemistry^8–10^, chemical vapor deposition (CVD) method^2, 3^ and welding techniques^11, 12^. The template  method is another approach to fabricate 3D porous carbon nanomaterials with a tunable pore size, large surface areas and interconnected pore network^13, 14^. All these methods rely on two main strategies; the first one is self-assembly method^15, 16^ and the second one is based on covalently interconnected structures^3, 9, 17–19^. The self-assembly strategy relies on physical interactions between individual nanostructures and their additives via Van der Waals forces, π-π interactions, electrostatic interactions and hydrophobic interactions^20, 21^. In the second strategy, carbon nanostructures are interconnected via covalent bonding by crosslinking chemistry^9, 22^, CVD^2, 3^ and welding techniques^12^. Based on the properties of 3D-carbon structures, they can be applied to a broad range of applications^21–26^. Such porous 3D carbon nanomaterials with large surface area are promising candidates as electrodes for supercapacitors.

Novel electrode materials with superior properties are very desirable for improving the performance of supercapacitors. One of the approaches for improving performances of carbon electrodes is by doping with heteroatoms^27^. Even though a large number of heteroatom doped carbon-based materials were reported as electrode materials for supercapacitors, their performance still needs to be improved with novel porous materials with large surface area. Nowadays, using natural products for developing renewable materials for energy storage systems has become increasingly important.

One of the novel systems, which can be used to improve energy storage capability of such devices, is a composite of carbon nanomaterials and natural organisms. Nature provide numerous and exceptional opportunities for production of complex structural and multifunctional materials. Bacteria are inexpensive, ample, environmentally friendly natural systems with variety of morphologies such as coccus, vibrio, fusiform bacilli and star-shaped bacteria. These natural systems can be used as a bio-template to create 3D carbon nanomaterials with interconnected pore network, large surface area as well as hetero-atoms. So far, there are some reports of natural materials being used as energy storage devices^28–30^. For example, H-W. Shim *et al*. reported the fabrication of porous Co_3_O_4_ nanostructures using Bacillus subtilis bacteria for lithium-ion battery applications^29^ and later as electrodes for supercapacitors^30^.

**Figure 1:**
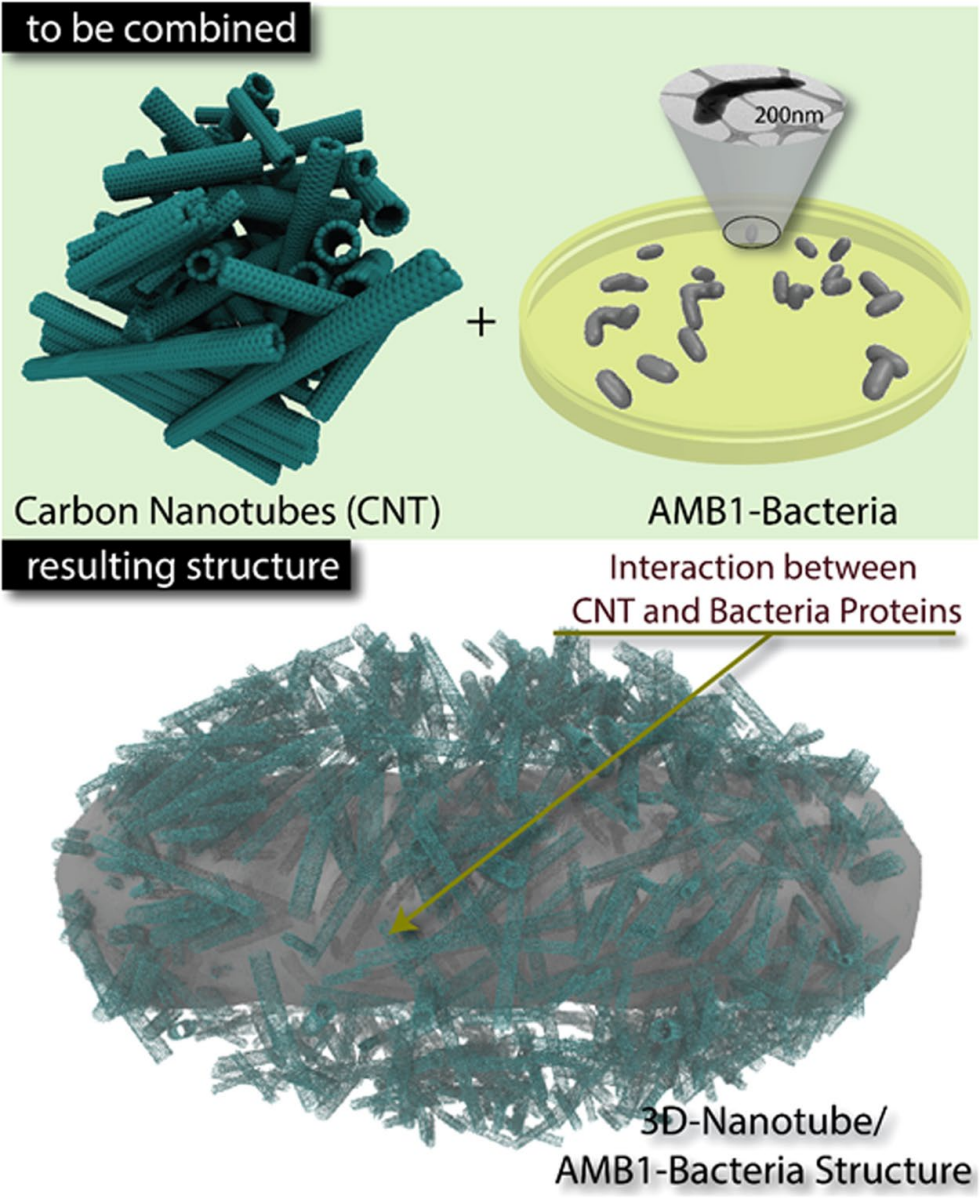
Schematic of the synthesis 3D CNT-AMB1 Bacteria composites. AMB1 bacteria behaves as a natural template for creating 3D hierarchical CNT-AMB1 macrostructure The non-covalent interaction between CNTs and AMB1 surface protein, MSP1, and flagellum protein, flagellin.

Here we report the fabrication of hierarchical 3D-CNTs structures with interconnected porous networks and large surface area by using Magnetospirillum magneticum (AMB-1) bacteria as a bio-template (Fig. 1). The resulting solid architecture is demonstrated as an electrode for supercapacitor applications because of the porosity, large surface area and heteroatoms present in the bacterial structure itself. AMB-1 is a class of magnetotactic bacteria that consists of intracellular magnetite nanoparticles called magnetosomes that aid them in controlling their direction under an external magnetic field. In addition of magnetite nanoparticles, AMB-1 has hetero-atomic structures in the form of surface protein MSP-1, which comprises 80% of the cell surface proteins, and flagellum protein, flagellin^31^. Combination of heteroatom structures, interconnected porosity and large surface area of carbon nanomaterials improve the charge transfer between electrode materials and ions of the electrolyte. The nature of binding between CNTs and the cell surface protein (MSP-1) and the outermost domain (D3) of the flagellum protein, flagellin are studied through steered molecular dynamics. It is found that the nano-bio interface between proteins and CNTs provide a unique binding kinetics to synthesize such 3D nanocomposite materials.

## Results and Discussion

**Figure 2:**
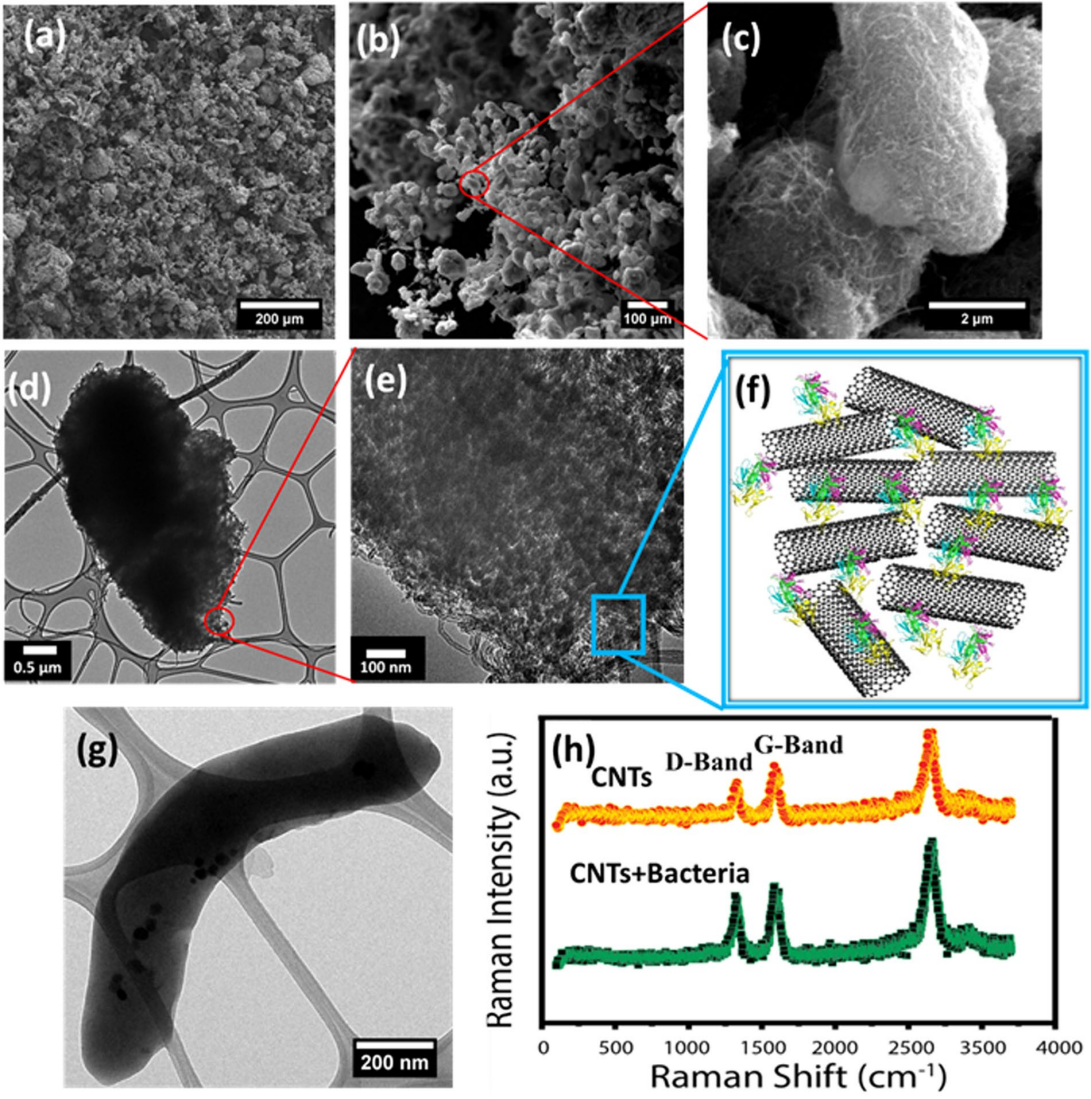
Microscopic and spectroscopic characterization of 3D nanotube/bacteria structure. (**a**–**c**) SEM images of CNT-bacteria structure shows that AMB1 bacteria acts as template for CNTs, (**d**–**e**) HRTEM images shows that nanotubes are integrated with bacteria proteins. (**f**) Schematic representative of interaction between CNTs and AMB1 surface proteins, MSP1 and flagellum. (**g**) The structural morphology of AMB1 bacteria (**h**) Raman spectra of CNTs and CNT-AMB1 structure.

To understand the morphology and structure of 3D CNT-AMB-1 architecture, detailed microscopy and spectroscopy studies have been performed. Scanning Electron Microscopy (SEM) has revealed that the morphology of 3D structure of CNTs-AMB-1 consists of interconnected porous structures (Fig. 2a). High-resolution SEM showed that the 3D macrostructure consists of microspheres/prolate (Fig. 2b). As it seems in Fig. 2c, bacteria structure (Fig. 2g) behaves as a bio-template because of the size dimensions and physical interactions such as hydrogen-bonding, π-π and Van der Waals interactions between AMB-1 bacteria and nanotube structure. High resolution transmission electron microscopy (HRTEM) has been performed in order to analyze the interactions between proteins of AMB-1 (Flagellin and MSP1) and nanotubes. CNTs are seen organized in a network primarily because of the non-covalent interactions between nanotubes and the proteins of AMB-1 such as flagellin and MSP-1 (Fig. 2d–f). The Raman spectra of the CNTs and 3D CNT-AMB-1 structure are shown in Fig. 2h. Raman spectra of nanotubes mainly consist of two main peaks: the first peak reveals the graphitic layer (G-band) that is closely related to the sp^2^ vibrations of the nanotubes. The second peak (D-band) comes from the disorder on the surface of CNTs^32, 33^. The Raman spectra of the pure nanotubes and 3D CNTs-AMB-1 structure show D band and G band at 1350 cm^−1^ and 1580 cm^−1^ respectively. A quantitative measurement of the defect density in the CNT sidewall can be determined by the ratio of these two bands, I_D_:I_G_, which does not show a significant change after the formation of the 3D CNT-AMB-1 structure. This indicates that the physical interactions between proteins and nanotubes does not give rise to any defects on the surface of CNTs. According to XPS studies, C1s core level peak positions of the carbon atoms are around 285 eV and the peak position of oxygen is about 532 eV, both with and without AMB-1; however, the N1s peak appeared approximately at 400 eV only after the CNT-AMB-1 structure is formed (Figure S1). In high resolution XPS characterization of the nanotubes, the C=C peak appears at 284.2 eV and the peaks at 284.9 eV and 286.3 eV demonstrate the presence of C-O and C=O functional groups, respectively. The C=C, C-N, C-O and C=O peaks changed to around 283.8 eV, 284.2 eV, 284.7 eV and 286 eV respectively after the combination of CNTs and AMB-1 (Figure S2) because of the physical interactions of AMB-1 and nanotube structures. The BET surface area (S_BET_) of freeze-dried CNTs solid is found to be 166 m^2^/g. The surface area of CNT-AMB1 solid is found to have been increased to 236 m^2^/g mainly because of the expansion between individual nanotubes and hence the increased surface area of nanotubes compared to pure nanotubes. DFT method for pore size distribution and cumulative pore volume of pure nanotubes and CNTs-AMB1 structures is shown in Figure S3b and c indicating that the pore size and the pore volume increased with the addition of the bacteria. The cumulative pore volume of CNTs-bacteria structure and pristine nanotube foam is 0.36 cm^3^/g and 0.18 cm^3^/g respectively (Figure S3c).

**Figure 3:**
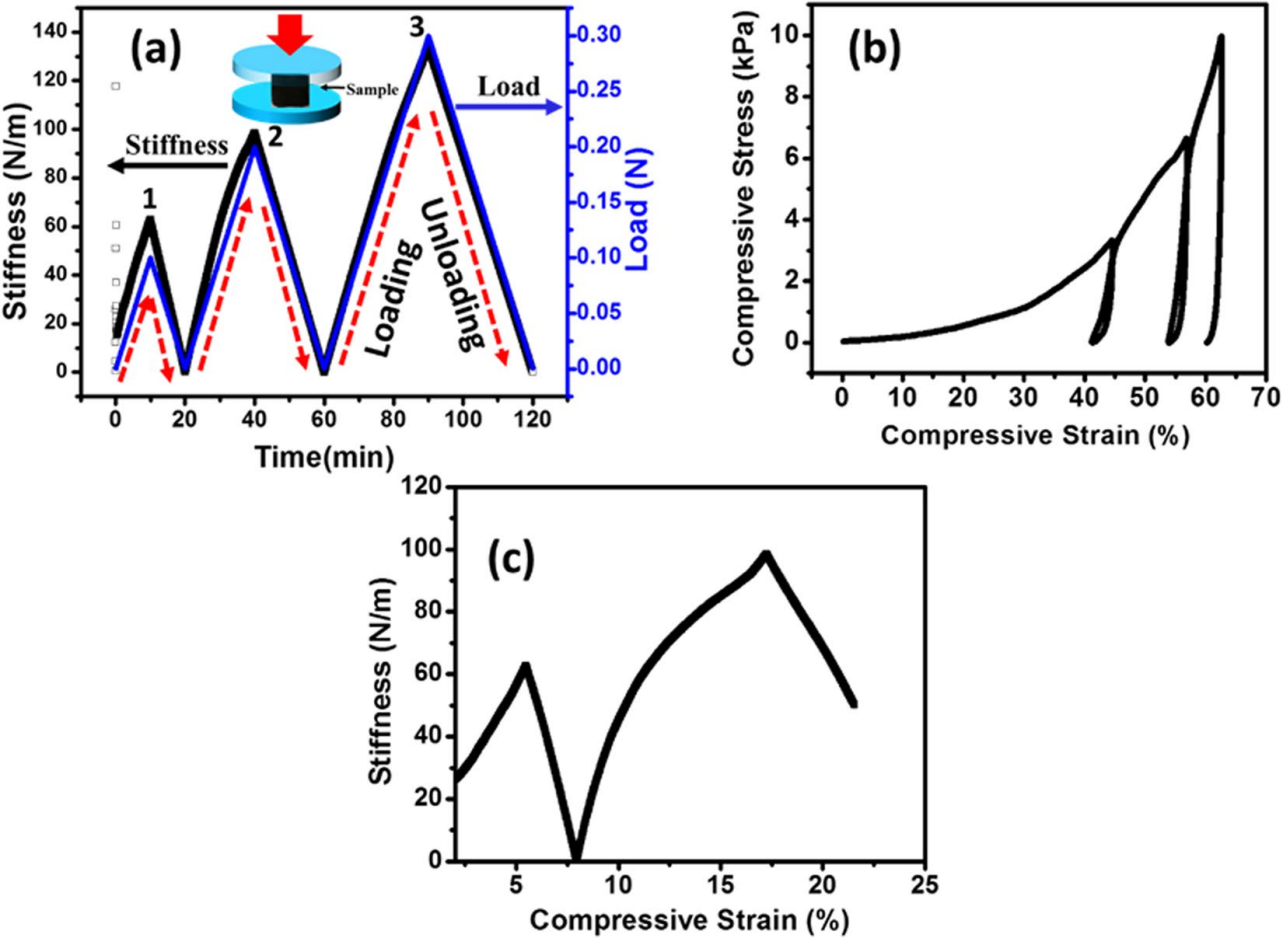
Mechanical characterization of 3D CNTs-Bacteria structure. (**a**) Load control experiment: Stiffness variation with three different cyclic loads (1:10, 2:40, and 3:90 N), (**b**) compressive stress-strain curve, (**c**) Variation of stiffness under compressive loading-unloading at different strain.

Compressive mechanical properties of CNTs-AMB-1 structure were measured with a dynamical mechanical analyzer (DMA). Figure 3a shows load–unload tests of 3D CNTs AMB-1 bacteria structure. The test was performed by varying the load while keeping all other variables constant. While the load on the 3D structure increases, the stiffness also increases. During loading as well as unloading the nature of the curve remains the same. This shows that the material is completely recoverable. These results show a maximum stiffness of 140 N m^–1^ at a load of 0.3 N. Figure 3b shows the load-unload stress-strain curves of 3D CNTs-AMB-1 bacteria foam. The compression loading curves indicate that the sponges can be compressed down to about 65% strain. As the load on the material increases, the nature of the curve remains the same during load-unloading test (Fig. 3b). The compression stress of the 3D CNTs-AMB-1 solid is about 10kPa at the strain of ~65%. Analysis of the Fig. 3b further shows that unloading curve does not recoil to the primary point. This is indicative of irreversible buckling of the cell wall with the porous structure of the foam. Furthermore, the compressive load-unload test in Fig. 3b reveals hysteresis within the foam which can be due to internal friction of the cell walls and viscoelasticity. To shed more light on the high compressive strain sustained by the foam without structural failure, we conducted a strained controlled test where strain is varied with stiffness. As shown in Fig. 3c, stiffness increases with increase in strain. Such an increase in stiffness with strain can be caused by enhanced entanglement between the nanotubes and the bacteria structure. Consequently, this entanglement makes it hard for the cell wall to buckle or break at the specified strain up to 65% as mentioned earlier.

**Figure 4:**
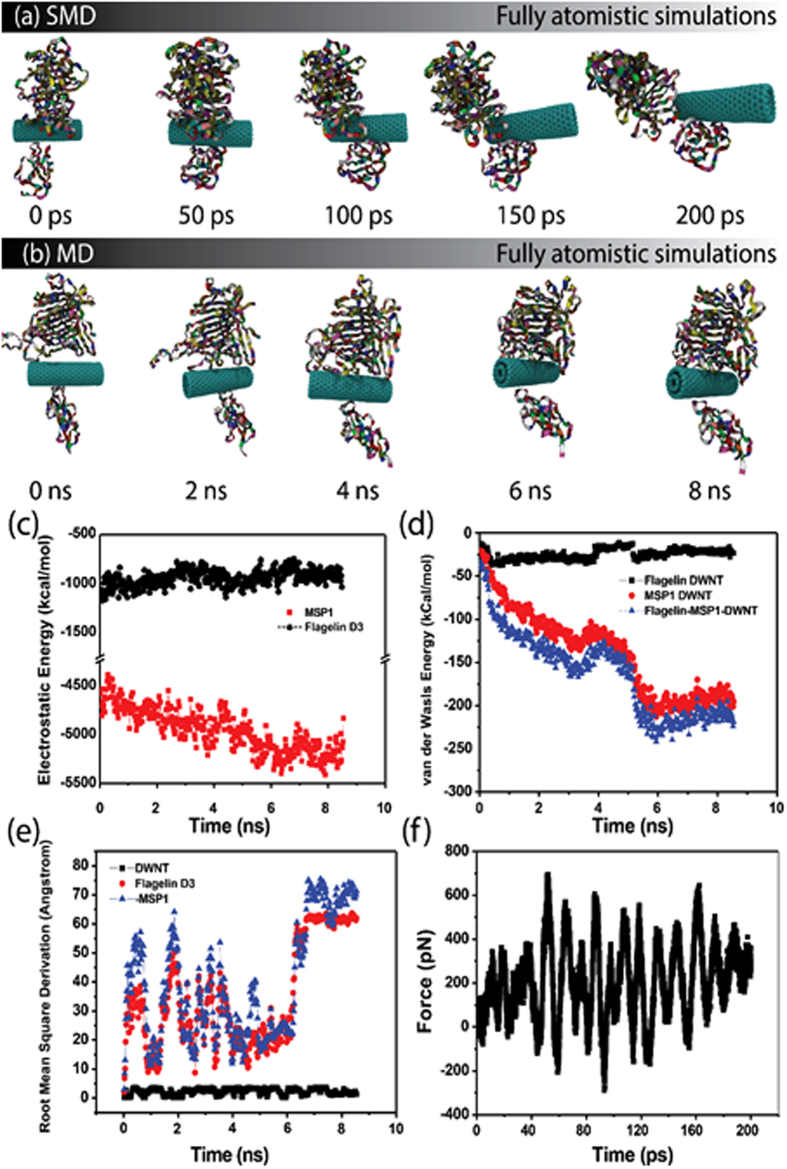
Interactions between CNT and AMB-1 via surface protein, MSP1 and flagellum protein, flagellin: (**a–b**) All atom simulation trajectory screenshots for steered molecular dynamics and protein adsorption onto the DWNT; (**c**) Electrostatic energy between DWNT and the surface protein, MSP1 and flagellin domain D3 showing five times larger interaction for MSP1 compared to D3, (**d**) Van der Waals interactions between DWNT and the proteins MSP1 and D3 indicating a larger VDW interactions between DWNT and MSP1 compared to D3, (**e**) Root Mean Square Deviation (RMSD) showing the adsorption of the proteins at ~7 ns onto the DWNT surface, (**f**) Force vs Time plot showing the presence of repulsive forces between the DWNT and the proteins indicating the role of these proteins and hence AMB1 as a crosslinker molecule for 3D CNT scaffold.

To study the interaction between the CNTs and the proteins from AMB-1, namely, the surface protein, MSP1, and the flagellum protein, flagellin, both molecular dynamics and steered molecular dynamics simulations were done (Fig. 4a). An all-atom simulation (~100,000 atoms) was performed for a time period of ~8.5 ns for a system containing DWNT (Double-walled carbon nanotube) with a diameter of ~1.6 nm in the presence of MSP1 and the outermost domain (D3) of flagellin (Fig. 4b). It was found that 8.5 ns was a good enough time period for favorable non-binding interactions between DWNT and both MSP1 and D3 proteins as seen from a relatively stationary RMSD (Root Mean Square Deviation) pattern that was noted for D3 (~60 Å) and MSP1 (~70 Å) after 7 ns (Fig. 4e). The non-binding energy of interaction was also analyzed and it was found that MSP1 does interact electrostatically (~5,250 kCal/mol) with DWNT, whereas the electrostatic interaction of D3 is relatively weak (~1,100 kCal/mol) (Fig. 4c). It is further found that the interaction energy is also governed by Van der Waals (VDW) forces largely for MSP1 but not so much for D3 as seen from Fig. 4d and that MSP1 interacts with DWNT with a VDW energy of ~200 kCal/mol, whereas D3 interacts with a relatively lesser VDW energy of ~30 kCal/mol. After a favorable adsorption was accomplished, steered molecular dynamics was utilized in a constant velocity mode (0.25 Å/ps, k = 7 kCal/mol/Å^2^) for 200 ps to pull the DWNT out from between the proteins (Fig. 4a). While the DWNT was being pulled, the dissociation energy was analyzed and plotted in the form of Force (pN) versus time (ps). It is seen from Fig. 4f that initially the applied attractive (snatching) force (maximum peak of ~300 pN) tries to overtake the force exerted by the proteins onto the DWNT (in terms of electrostatic and VDW energies, with a peak of ~700 pN). However, it is seen that even after 200 ps, there is a significant repulsive force (~200 pN) still left that the proteins exert electrostatically (because the distance between the proteins and the DWNT is no longer within the VDW range) indicating that there exists a strong interactive energy to bind these proteins (and hence, AMB-1) with the carbon nanotubes. It is because of this strong repulsion to dissociation from the AMB-1 that allows this CNT based nanocomposite to become conducive to 3D architecture.

**Figure 5:**
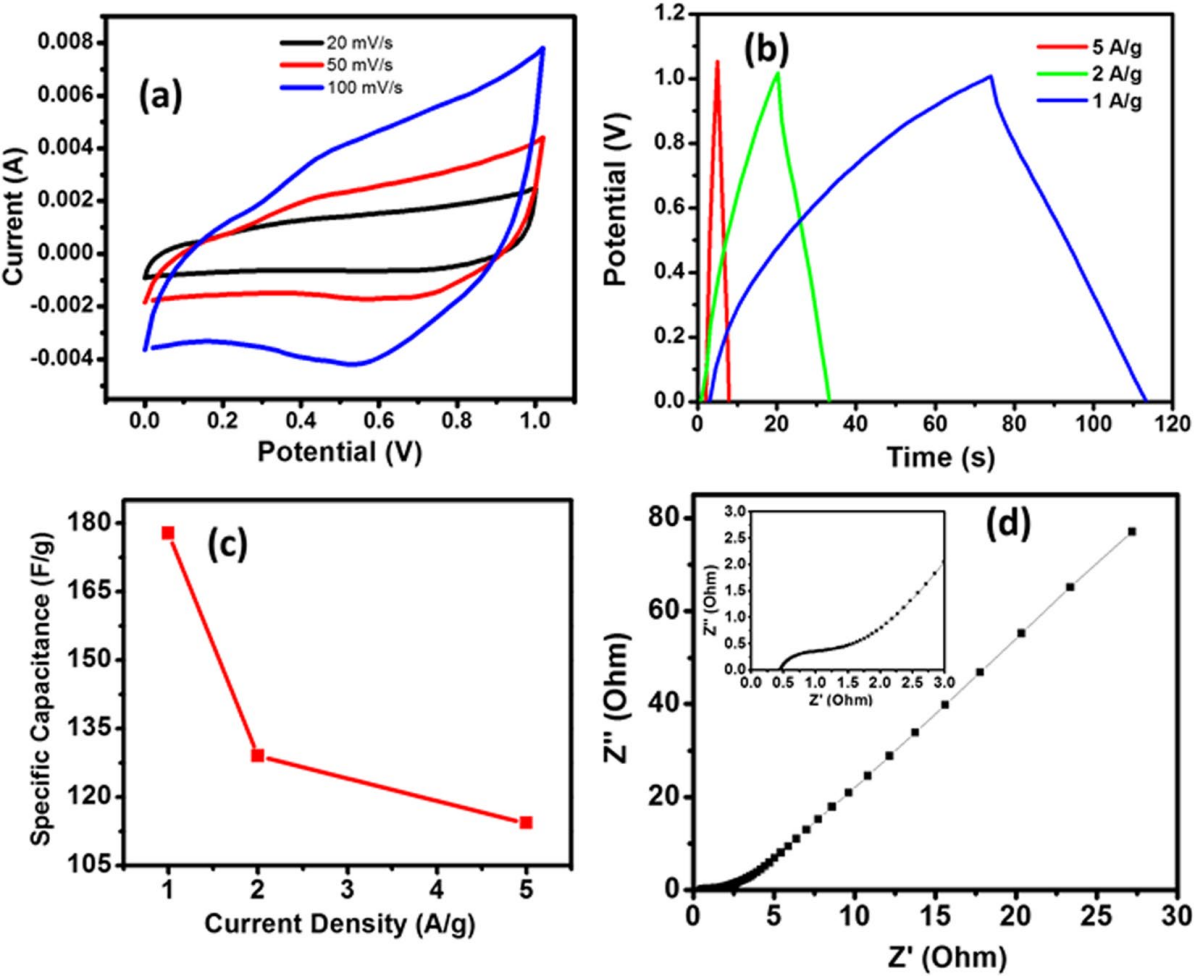
Electrochemical measurement of 3D CNTs-Bacteria structure, (**a**) CV curves of 3D CNT-Bacteria electrode at different scan rates, (**b**) Galvanostatic charge/discharge curves of 3D CNT-Bacteria structure at different current density (**c**) Variation of specific capacitances of the CNTs-Bacteria electrodes with current density, (**d**) Nyquist impedance plots of the 3D CNT-Bacteria electrode.

The electrochemical characterization of pure CNTs is shown in Figure S5. The cyclic voltammetry (CV) curve of CNTs that is measured by a two-electrode system with a symmetric supercapacitor in 1.0 M Na_2_SO4 aqueous electrolyte (Figure S5a). The galvanostatic charge/discharge curves of the CNTs structure in 1 M Na_2_SO_4_ aqueous solution were performed at a current density of 1, 2 and 5 A/g. All the curves unveil a triangular shape showing that the CNTs structure has a high reversibility of the charge/discharge process (Figure S5b). The highest﻿ specific c﻿apacitance of pure CNTs is 28 F/g﻿. Figure 5 shows electrochemical characterization of 3D nanotube-bacteria structure Fig. 5a shows that the cyclic voltammetry (CV) curve of CNT-AMB-1 composite, which is evaluated using a two-electrode system with a symmetric supercapacitor in 1.0 M Na_2_SO_4_ aqueous electrolyte. The galvanostatic charge/discharge curves of the pure CNTs and 3D CNTs-Bacteria structure in 1 M Na_2_SO_4_ aqueous solution were carried out at a current density of 1, 2 and 5 A/g. As shown in Figs 5b and S5b, all the curves exhibit a triangular shape, which indicate that the 3D CNT-AMB-1 structure has a high reversibility of the charge/discharge process. The charging and discharging processes take longer time at lower current density because of the insertion or release of the amount of Na ions during these processes. The specific capacitance of the 3D CNT-AMB-1 structure at different current density is shown in Fig. 5c. The capacitance of the structure is 177.8, 129.2 and 114.3 F/g at 1, 2 and 5 A/g respectively. We assume that magnetite and other heteroatoms in the bacterial structure play a crucial role for the charge storing capacity of the 3D composite structure. Additionally, the increased surface area, 236 m^2^/g is another important factor that affect the capacity of the CNT-AMB-1 structures while the surface area of CNTs is 166 m^2^/g. This bacteria templated CNT-based supercapacitor has showed 6 fold  higher specific capacitance compared to pure CNTs﻿. To understand the electrochemical performance of the 3D CNT-AMB-1 electrode, the electrochemical impedance spectroscopy (EIS) was conducted (Fig. 5d). For an ideal double-layer supercapacitor, the impedance plot should be a vertical line parallel to Z” axis and in general this behavior is observed in CNT electrodes. As it can be seen in Fig. 5d, Nyquist plots show a good capacitive behavior, as indicated by the near vertical line over the low-frequency ranges. The abrupt increase of the imaginary part of EIS at lower frequency is because of the capacitive behavior of 3D CNTs-Bacteria electrode. In the inserted figure in the Nyquist plot, the semicircle loop at lower frequencies shows charge-transfer process of the electrode. SEM characterization of CNT-bacteria composite is performed before and after electrochemical characterization to understand the structural evaluation of the electrode (Figure S6). As seen in figure S6, the structure of composite is nearly same before and after the electrochemical test.

## Conclusion

In conclusion, we report renewable and scalable light-weight 3D porous macrostructure using CNTs and Magnetospirillum magneticum (AMB-1) bacteria. The resulting porous scaffold showed good mechanical stability. To understand the interactions between CNTs and AMB-1 bacteria’s cell surface protein MSP-1 and flagellum protein flagellin, steered molecular dynamics simulations were used, which are in good agreement with the experimental data. The 3D CNTs-AMB-1 nanocomposite scaffold is further demonstrated as an electrode for supercapacitor applications. The highest capacitance of light-weight, porous structure is 177.8 F/g, which improved more than six times compared to pure CNTs. We propose that magnetite and heteroatoms of the bacterial structure have an important role for the charging and discharging capacity of the 3D CNT-AMB-1 structure.

## Method

Carbon nanotubes were obtained from cheaptubes.com (Outer Diameter: 20–30 nm, length 10–30 µm). M. Magneticum AMB-1(The average size of bacteria: 5–8 µm in length, 500 nm diameter) was purchased from the American Type Culture Collection (ATCC 700264) and microaerobically cultured in magnetic spirillum growth medium as previously described by Matsunaga *et al*.^34^. Before autoclaving, 280 ml of the media was divided into 20 test tubes (14 ml each) and 0.001 g of CNTs was added to each test tube. Once sterilization was done, 0.5 ml of AMB-1 (~10^6^ cells per ml) culture was inoculated per test tube and incubated at 28 °C for one week. After one week, the functionalized CNTs were harvested by centrifuging the cultures at 7000 rpm for 30 minutes. The pellets were then allowed to dry overnight. Cell density is 0.32 g/L, which typically start with a concentration of 10^6^ cells/ml and reach ~2 × 10^8^ cells/ml at the end of the culture cycle. MWNTs density is 0.084 g/L. We added 1 mg of MWNTs per 12 ml of media along with 0.5 ml of cell culture per test tube. Raman spectroscopy (633 nm laser), X-ray photoelectron spectroscopy (XPS), scanning electron microscope (SEM) (SEM, FEI Quanta 400 ESEM FEG), and transmission electron microscope (TEM) (JEOL 2100 Field Emission Gun TEM) were used for structural characterization of the materials. Mechanical properties of the 3D structure were tested by Dynamical Mechanical Analyzer (DMA). For the electrode fabrication for supercapacitor measurements, CNT and CNT-bacteria (70 wt%) was further mixed with conducting carbon (20 wt%) and polyvinylidenefluoride (10 wt%) as binder using N-methyl-2-pyrrolidone as solvent. The slurry thus obtained was casted on copper foil. The sandwich type cell configuration where two symmetric electrodes were separated using 1MNa_2_SO_4_ electrolyte soaked filter paper. The coin cell thus prepared were tested by cyclic voltammetry measurement and galvanostatic charge discharge at AUTOLAB PGSTAT 302 potentiostat.

## Electronic supplementary material

Bacteria as Bio-Template for 3D Carbon Nanotube Architectures
