# Supplementary material for: Bacteria as Bio-Template for 3D Carbon Nanotube Architectures
Source: Sci Rep. 2017 Aug 29;7:9855. doi: 10.1038/s41598-017-09692-2 (PMC5575067; doi:10.1038/s41598-017-09692-2)
Supplement: Supplementary file 1 — Bacteria as Bio-Template for 3D Carbon Nanotube Architectures [file 41598_2017_9692_MOESM1_ESM.pdf]

Supporting Information

Bacteria as Bio-Template for 3D Carbon Nanotube Architectures

Sehmus Ozden[1], Isaac G. Macwan[2], Peter S. Owuor[3], Suppanat Kosolwattana[3], Pedro A. S. Autreto[4], Sushila Silwal[2], Robert Vajtai[3], Chandra S. Tiwary[3], Aditya D. Mohite[1], Prabir K. Patra[2], Pulickel M. Ajayan[3].

[1] Materials Physics and Applications Division, Los Alamos National Laboratory, Los Alamos NM, 87545, USA

[2]Department of Biomedical Engineering, University of Bridgeport, 126 Park Avenue, Bridgeport, CT

[3] Department of Material Science and NanoEngineering, Rice University, Houston, Texas 77005, USA

[4]Universidade Federal do ABC, Santo André-SP, 09210-580, Brazil

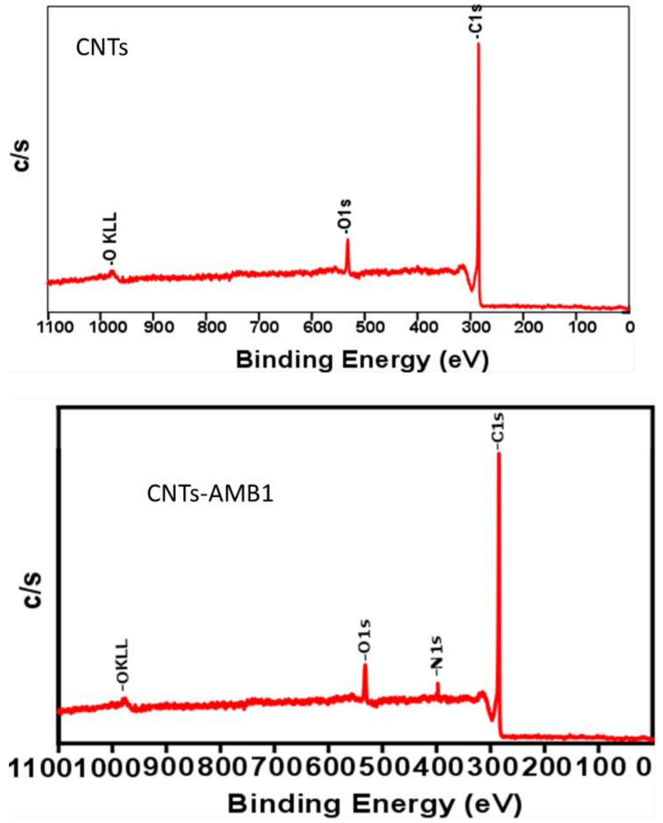

Figure S1. Survey scan XPS characterization of CNTs and CNTs-AMB1 bacteria.

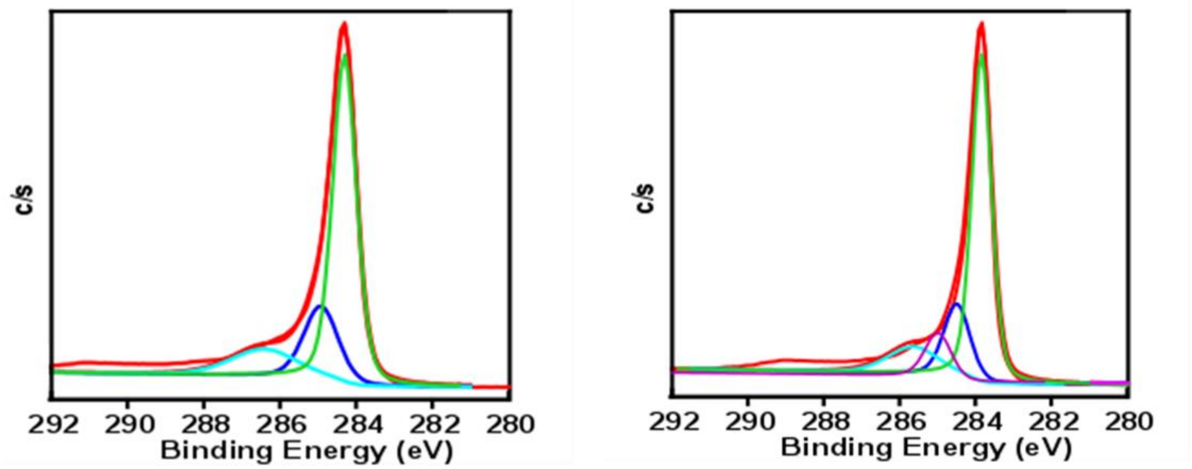

Figure S2. High resolution XPS characterization of CNTs and CNTs-AMB1 bacteria.

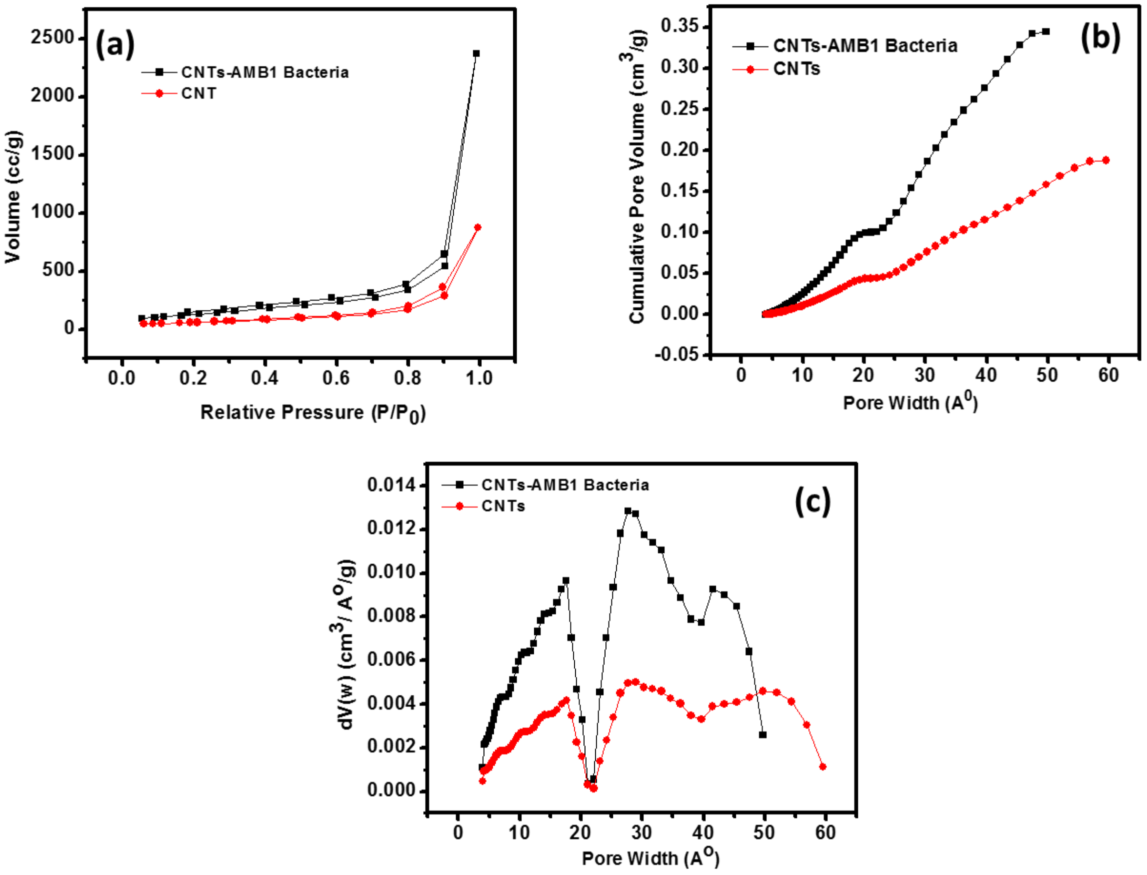

Figure S3. . a) The N_2 absorption isotherm of CNTs and 3D CNTs-Bacteria structure (b) Cumulative pore volume as a function of pore size, (c) DFT method pore size distribution of CNTs and 3D CNTs-Bacteria structure.

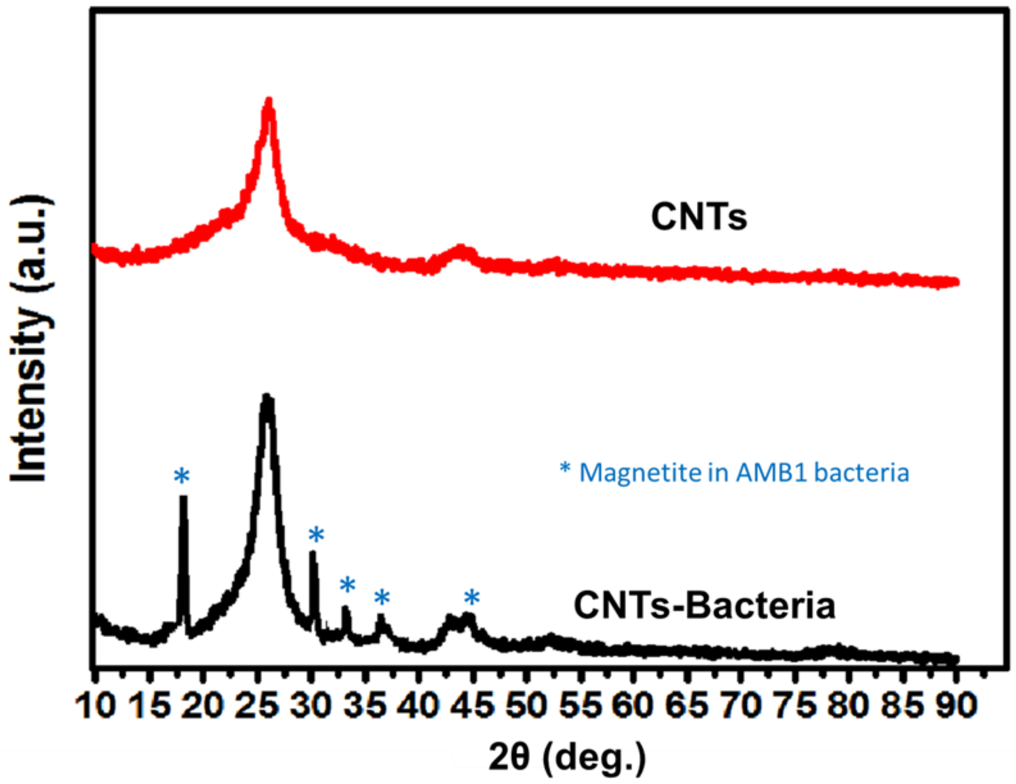

Figure S4. XRD characterization of CNTs and CNTs-AMB1 bacteria.

Compared to pure CNTs, the CNTs-AMB1 bacteria results showed some additional peaks, which are belong to magnetite in AMB1 bacteria (ref: J. R. Soc. Interface (2011) 8, 1011–1018, doi:10.1098/rsif.2010.0576)

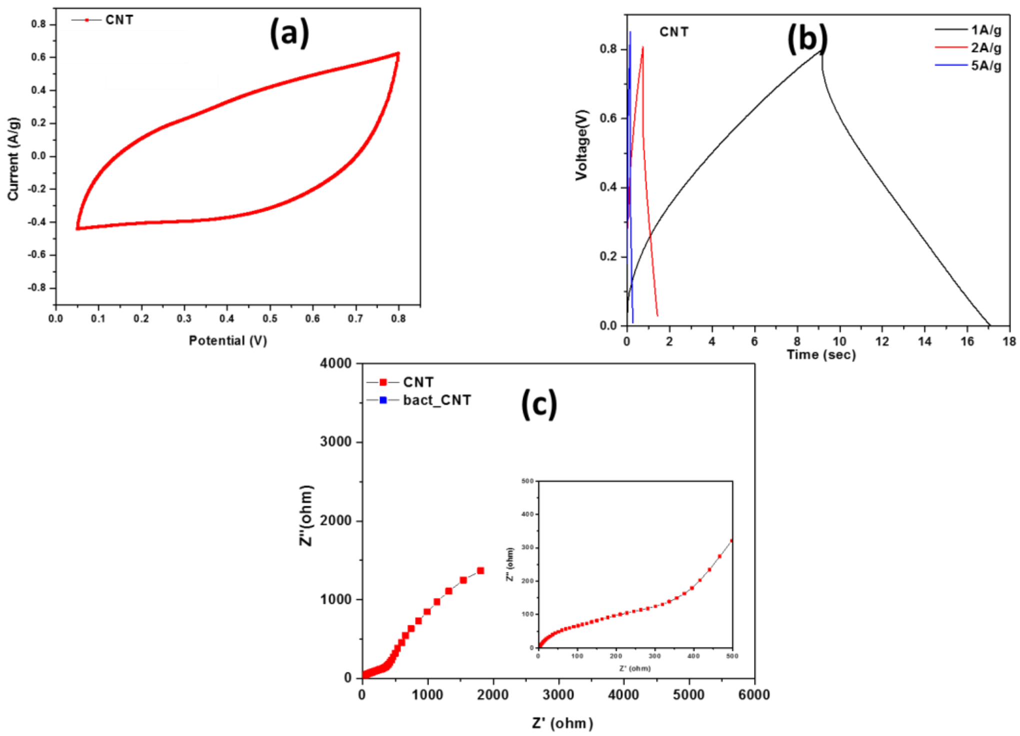

Figure S5. Electrochemical measurement of CNTs (a) CV curves of CNT electrode at 100 mV scan rate, (b) Galvanostatic charge/discharge curves of CNTs structure at different current density (c) Nyquist impedance plots of the CNTs electrode.

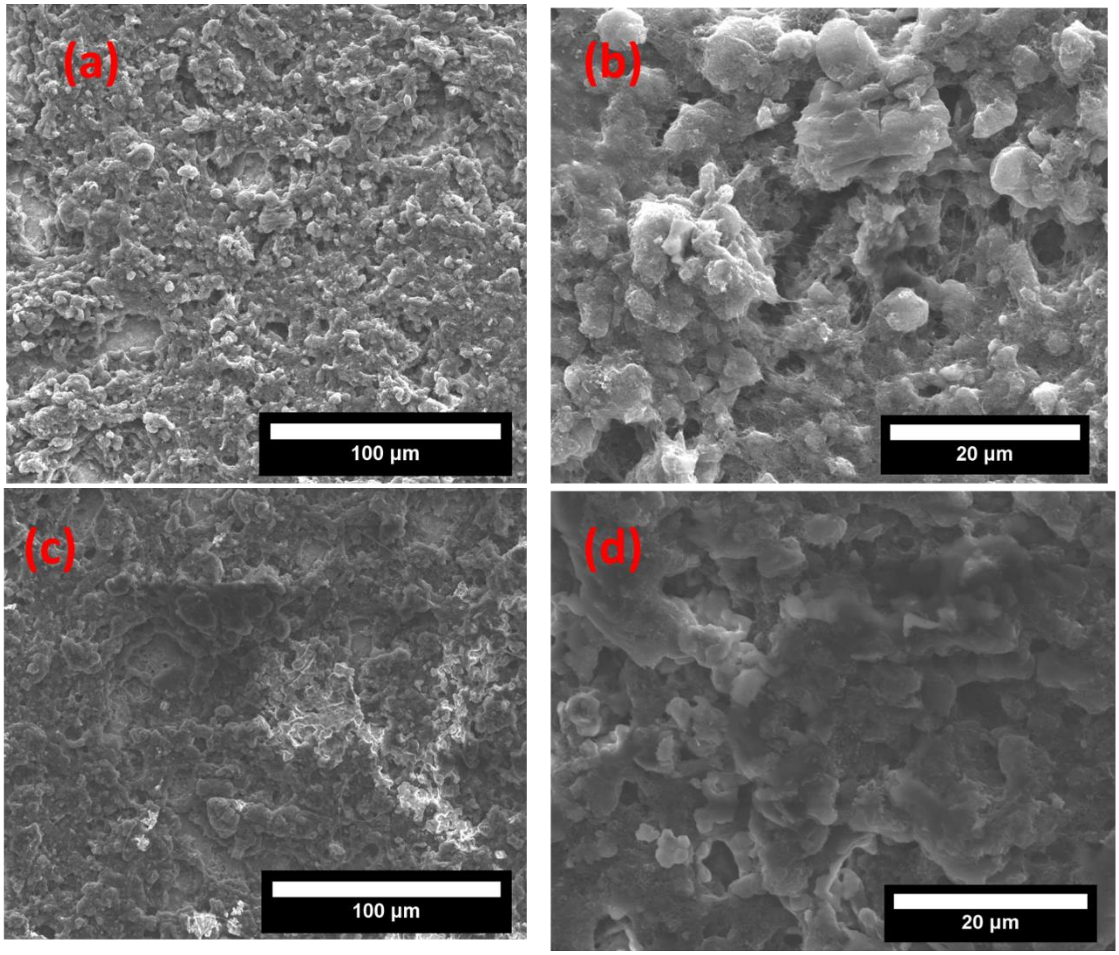

Figure S6. SEM characterization of CNTs-AMB1 bacteria (a-b) before and (c-d) after electrochemical measurement.

The cyclic voltammetry (CV) curve of CNTs that is measured by a two-electrode system with a symmetric supercapacitor in 1.0 M Na_2SO_4 aqueous electrolyte (Figure S5a). The galvanostatic charge/discharge curves of the CNTs structure in 1 M Na_2SO_4 aqueous solution were performed at a current density of 1, 2 and 5 A/g. All the curves unveil a triangular shape that show that the CNTs structure has a high reversibility of the charge/discharge process (Figure S5b).
